# Radio Electric Asymmetric Conveyer (REAC) Reparative Effects on Pressure Ulcer (PU) and Burn Injury (BI): A Report of Two Cases

**DOI:** 10.7759/cureus.27060

**Published:** 2022-07-20

**Authors:** Vania Fontani, José Alfredo Coelho Pereira, Margarete Carréra Bittencourt, Salvatore Rinaldi

**Affiliations:** 1 Research Department, Rinaldi Fontani Foundation, Florence, ITA; 2 Department of Regenerative Medicine, Rinaldi Fontani Institute, Florence, ITA; 3 Escola de Enfermagem Magalhães Barata, Universidade do Estado do Pará, Belem, BRA

**Keywords:** radio electric asymmetric conveyer, biostimulation, regenerative medicine treatments, burn injury reparative medicine treatments, pressure ulcer

## Abstract

Pressure ulcer (PU) and burn injury (BI) represent two types of wounds that require broad and difficult fields of treatments. Despite various advances made in recent years, these injuries have few solutions that allow recovery in shorter times and with greater effectiveness. All this negatively affects the patient's quality of life. Since ancient times, with the use of torpedoes (a kind of fish capable of producing electric discharges), it has been believed that the use of electricity could favor the repair processes of various kinds of wounds.

Today, technological evolution has allowed the creation of more and more advanced techniques that can determine a better reparative response of the injured tissues. The radio electric asymmetric conveyer (REAC) technology is one of these and the reparative tissue optimization (TO-RPR) treatment represents the specific treatment for these lesions. The two cases presented in this article are intended to highlight how two serious injuries of a different nature, when treated with the REAC TO-RPR, have the same rapid qualitative and quantitative recovery path that continues even after the end of the treatment cycle. The stability and progression of the effects are typical of REAC treatments, and in this article, it is possible to appreciate the clinical evidence. These results together with others previously published open a new therapeutic possibility in the treatment of wounds.

## Introduction

A wound is generally considered to be a rupture in the integument of an organism produced by acute trauma or chronic processes, which include but are not limited to the following etiologies: venous ulcers, diabetic ulcers, and pressure ulcers (PUs), involving signs that trigger a multitude of biological phenomena aimed at restoring the functional integrity of tissues [1].

The wound itself is a sign of damage and, in still viable organisms, the resulting biological mobilization is aimed at repair, starting from an instantaneous physical event that is often overlooked, but that accompanies every wound, a dramatic change in the electrophysiological character of the intact skin surrounding the wound [2].

PU, a debilitating chronic wound, is a significant and still common problem in all health settings, affecting approximately 10% of hospitalized patients, especially in intensive care units [3,4].

PU is a usual problem especially in elderly patients with immobility and inactivity due to significant co-morbidities such as diabetes and arterial vascular disease, carrying very significant human and economic impacts [5,6].

PUs represent a major burden to patients, including negative psychological, physical, and social consequences affecting health, well-being, and health-related quality of life [7]. Effective healing of complex chronic wounds, such as PU, still represents a clinical challenge.

Burn injury (BI) represents another interesting chapter of vulnology [8]. BIs, if severe, always involve an inflammatory and immune response as well as metabolic changes that often have an influence on a systemic level to the point of determining multi-organ failure [9]. Like PUs, BIs can have a strong impact not only on physical health but also on the patient's mental health and quality of life [8]. BIs are classified as follows: First-degree burns, which affect only the epidermis or outer layer of skin; second-degree burns, which involve the epidermis and part of the dermis layer of skin; and third-degree burns, which destroy the epidermis and dermis [10]. Third-degree burns may also damage the underlying muscles, tendons, and bones.

Some radio electric asymmetric conveyer (REAC) technology treatments have demonstrated reparative and regenerative efficacy [11-13]. Among the tissue optimization (TO) treatments, the reparative type (TO-RPR) is aimed at promoting reparative effects in a non-invasive and painless way [13]. It is administered by covering the area to be treated with a planar probe, which constitutes the asymmetric conveyer probe (ACP) connected to the REAC device. When the area to be treated, for various reasons, such as bleeding, cannot be covered by the ACP, this can be positioned in the area opposite the lesion, making the treatment simpler and in any case effective.

The REAC technology was designed for neurobiological stimulation treatments through the reorganization of the endogenous bioelectric activity at the molecular level, according to precise therapeutic protocols, for the various indications of use.

The therapeutic effect is determined by the interaction of the radio electric field generated by the REAC device with the molecular structures of the tissues through the ACP connected to the device. This interaction optimizes the endogenous bioelectric activity. The effects are diversified according to the type of treatment protocol used. The TO-RPR type has been designed to promote reparative processes.

REAC TO-RPR treatments are treatments preprogramed for their intended use by the manufacturer of the REAC medical devices and cannot be changed in any way by the operator. There are no contraindications with the use of drugs of various nature and indications.

The REAC TO-RPR in these two reported cases was administered twice a day, for a total of 18 treatments of 15 minutes each.

The REAC model device used in this study was BENE 110 (ASMED, Florence, Italy).

## Case presentation

Case 1

The first case concerns a patient suffering from a PU. The patient is a 70-year-old Caucasian man, with a history of hypertension and diabetes for 12 years, having undergone angioplasty six years ago, presented with ischemic stroke with hemorrhagic transformation on January 16, 2020. Being submitted to decompression craniotomy plus duroplasty, he remained hospitalized for a period of two months, during which time he developed an extensive PU in the sacral region, despite the fact that he was on an anti-bedsore mattress.

**Figure 1:**
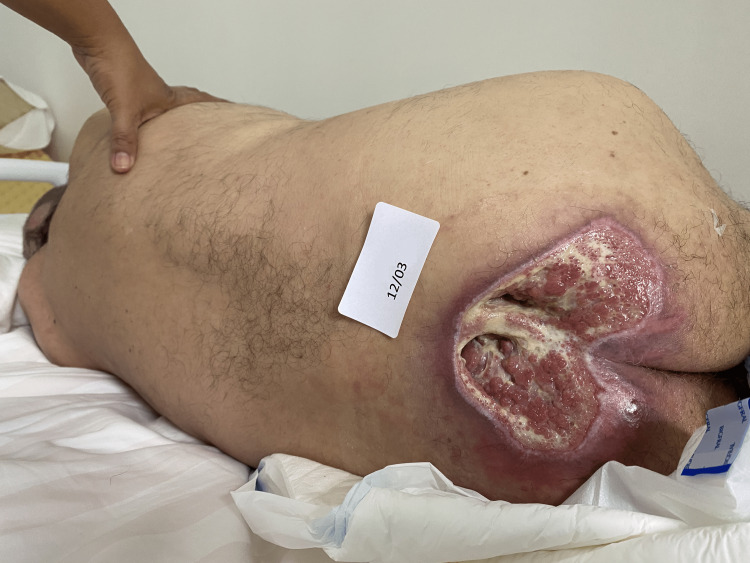
Situation of the pressure ulcer (PU) before treatment with radio electric asymmetric conveyer reparative tissue optimization treatment (REAC TO-RPR)

At our initial examination, on March 12, he presented a delimited, deep lesion, full thickness tissue loss with exposed muscle, erythematous borders, a bed filled with sloughs, moderate serous exudation, without odor, hemorrhage, and signs of infection, NPUAP/EPUAP Pressure injury classification Stage IV (Figure 1 and Video 1).

**Video 1:** Situation of the PU before treatment with REAC TO-RPR

On the same day as the first examination, the patient received the first REAC TO-RPR session.

**Figure 2:**
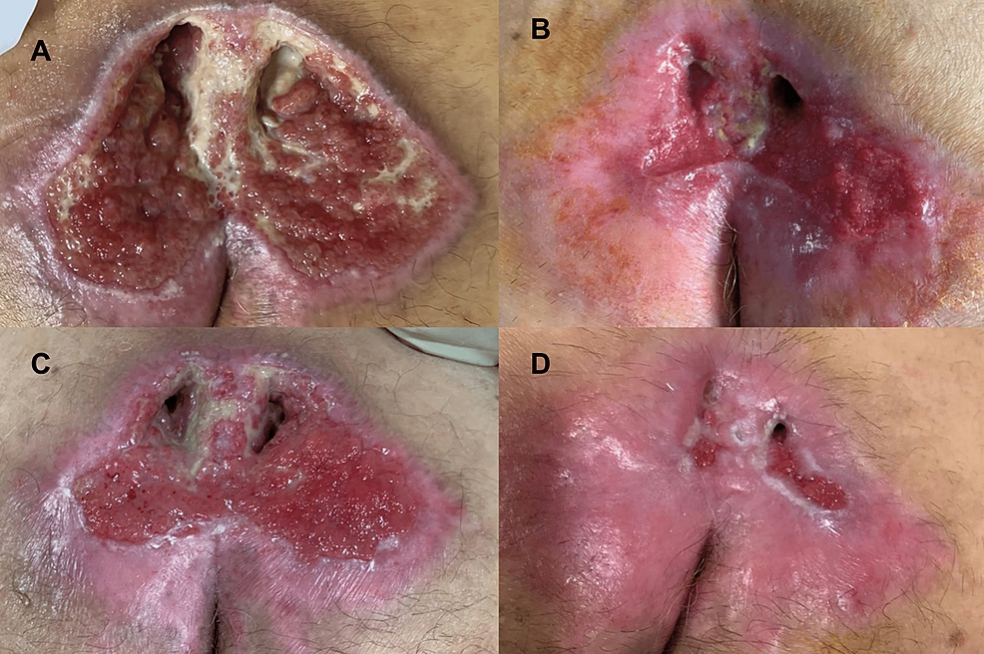
Evolution of the reparative process of the PU during treatment and after the end of the REAC TO-RPR treatment Figure 2A: Situation of PU at fifth day of treatment; Figure 2B: Situation of PU at six days from the end of the treatment; Figure 2C: Situation of PU at 10 days from the end of the treatment; Figure 2D: PU situation after six weeks from the end of the treatment

**Figure 3:**
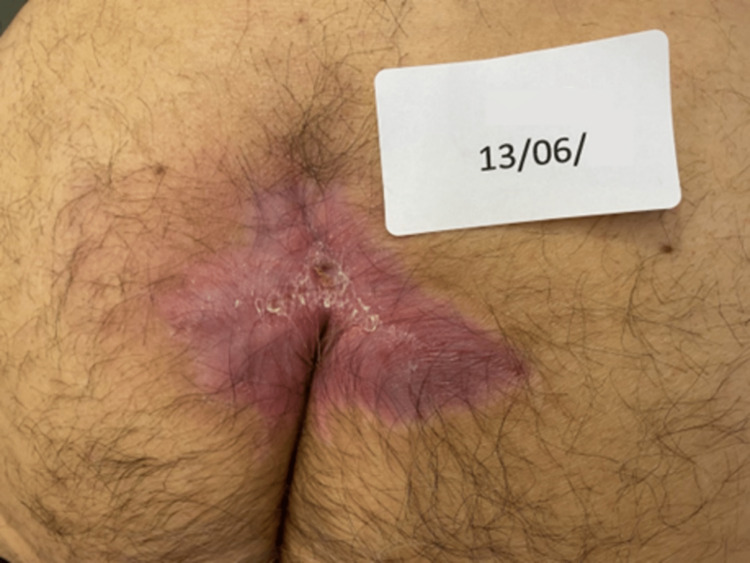
Complete PU healing three months after the start of the REAC TO-RPR treatment

Given the severity of the injury, and the patient's comorbidities, such as cardiopathy and diabetes, a long evolution with the possibility of serious clinical complications, would be expected, including infection, reaching a septic condition. On the contrary, after the REAC TO-RPR, there was an accelerated tissue repair process, reaching its resolution in a short time (12 weeks). In addition to the short healing time, a new hydrated tissue, without retraction stands out. The recovery of the tissue's physiological pattern and the refined reparative process are highlighted by the regrowth of skin appendages such as hair also on the scar surface, which is not observed in processes of this nature in conventional treatments (Figures 2 and 3).

Case 2

**Figure 4:**
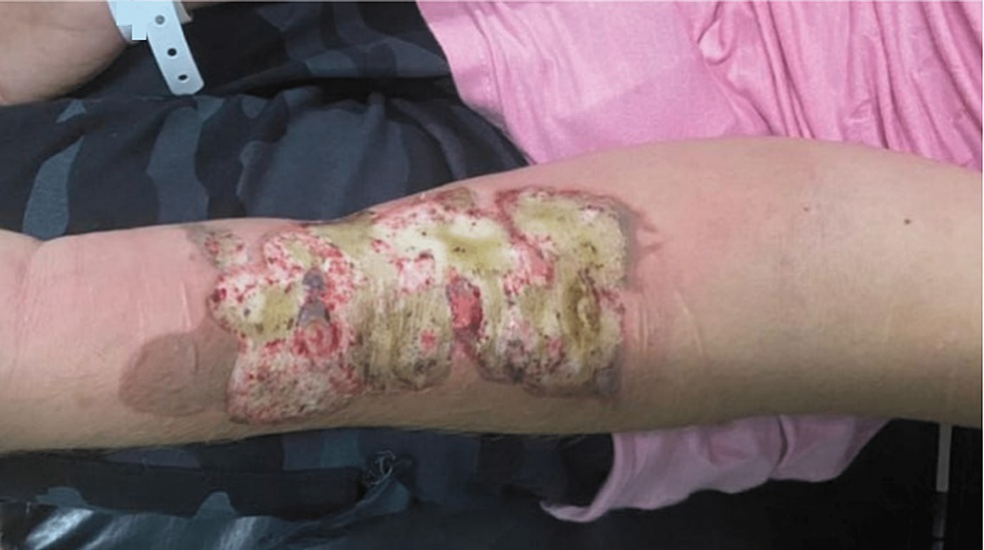
Initial status of the lesion at the time of admission of the patient to the Burn Treatment Center.

The second case concerns a man, 21 years old with a deep second-degree B). The extended BI was provoked by an act of self-harm. The subject in question set fire to the surface of a soup spoon and once it became incandescent, using its convex part, it began to produce lesions. The patient repeated this self-injurious act several times to cover the entire surface of the left forearm (Figure 4).

**Figure 5:**
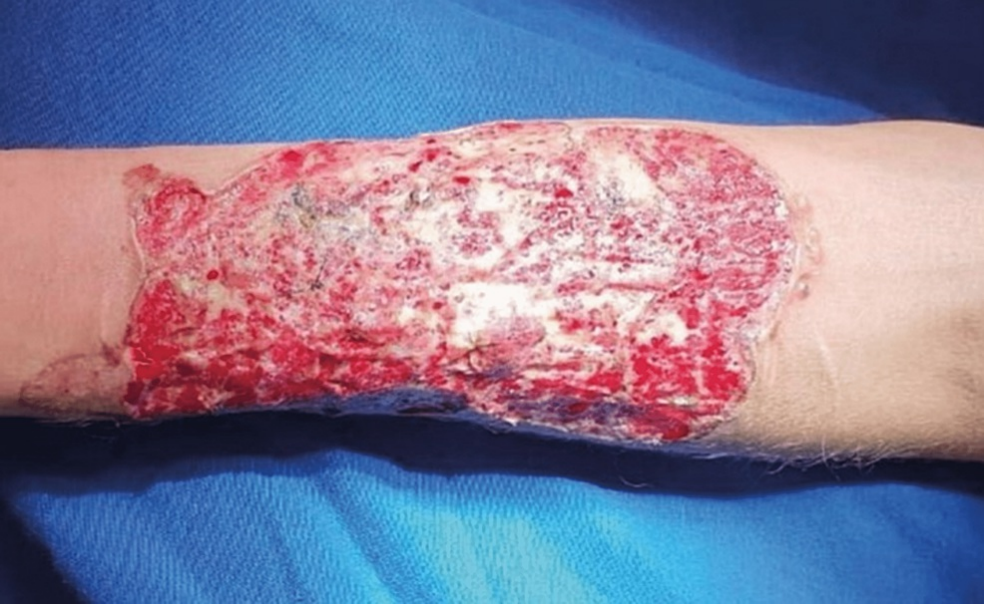
Situation of the lesion after the second debridement

Initially attended at the reference service Burn Treatment Center on June 14 after preliminary evaluation, he underwent a surgical procedure for tangential debridement to remove devitalized tissue. On the same day, he underwent a new tangential debridement for the removal of devitalized tissue (Figure 5).

**Figure 6:**
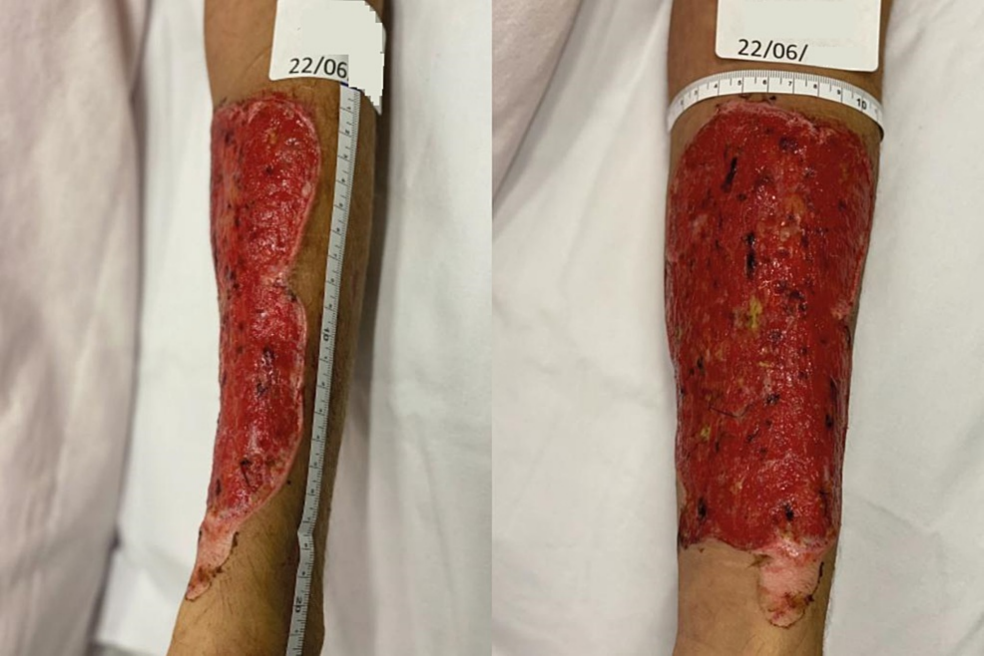
Situation of the burn injury (BI) at the time of our first medical examination

Since the patient had already been involved in similar acts several times, the doctors decided not to proceed with a skin graft transplant, and the patient was referred to our service. The patient's situation at the time of our first observation on 22 June is shown in Figure 6.

**Figure 7:**
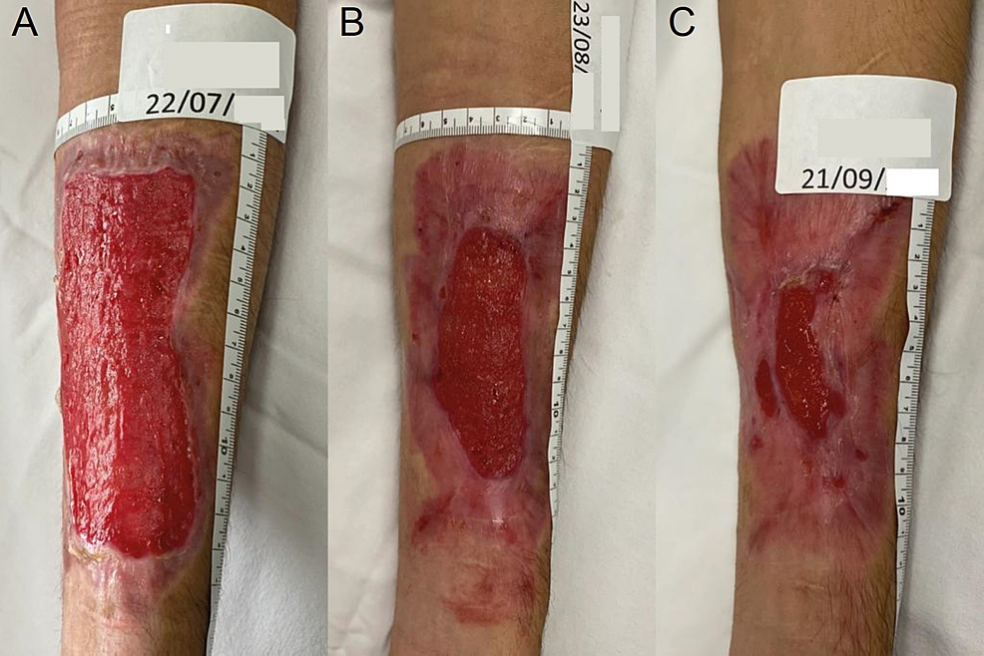
Evolution of the reparative process of the BI after the end of the REAC TO-RPR treatment Figure 7A: Situation of BI after one month from the start of REAC TO-RPR treatment; Figure 7B: Situation of BI after two months from the start of REAC TO-RPR treatment; Figure 7C: Situation of BI after three months from the start of REAC TO-RPR treatment

**Figure 8:**
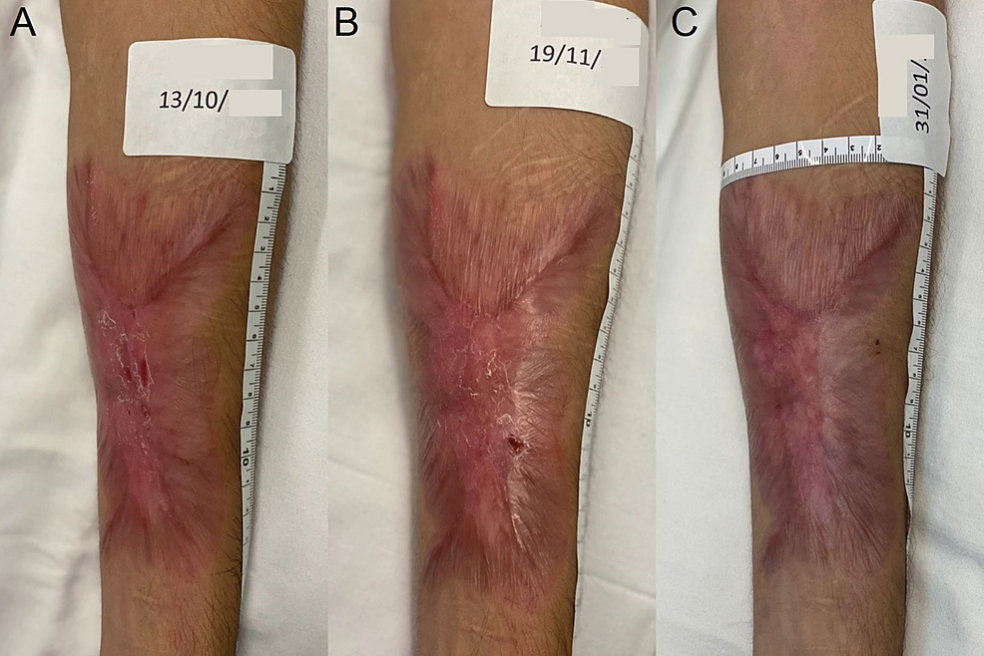
Evolution of the reparative process of the BI after the end of the REAC TO-RPR treatment Figure 8A: Situation of BI after four months from the start of REAC TO-RPR treatment; Figure 8B: Situation of BI after five months from the start of REAC TO-RPR treatment; Figure 8C: Situation of BI after seven months from the start of REAC TO-RPR treatment

In this case, we used the same cycle of 18 sessions of REAC TO-RPR treatment, each lasting 15 minutes. The positive and rapid evolution of the reparative processes of BI can be observed in Figures 7 and 8.

**Figure 9:**
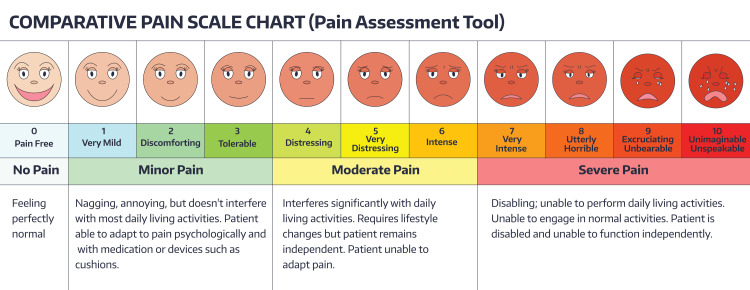
Visual analog scale Permission of usage obtained from GettyImages.

Since pain represents an important component in this type of injury, especially during handling for cleaning and daily dressings, we have decided to evaluate the perception and communication of pain using a visual analog scale, to be administered at each session of REAC TO-RPR treatment (Figure 9).

The perception and communication of pain trend during the REAC TO-RPR treatments are summarized in Table 1.

**Table 1:** Case 2, perception and communication of pain during treatment '-' indicates session not performed

TO-RPR-15	Morning		Evening	
Days	Before Session	After Session	Before Session	After Session
22 June	7	8	4	4
23 June	8	6	5	2
24 June	-	-	5	1
25 June	5	2	4	0
28 June	0	0	0	0
29 June	0	0	0	0
30 June	0	0	-	-
01 July	0	0	0	0
02 July	-	-	0	0
05 July	-	-	0	0
07 July	-	-	0	0

The results reported in Table 1 show how the reparative/regenerative processes have had an important impact on the perception and communication of pain. In fact, after the second session of the fourth day from the start of the REAC treatment, the patient reported no more pain. This result is totally unusual in other therapeutic approaches.

## Discussion

Regenerative medicine has always focused its attention fundamentally on chemical-biological tools, probably because they are potentially more easily produced and used on a large scale. All this probably had a negative impact on the promotion of the search for the therapeutic manipulation of endogenous ion flows (EIF). EIFs play a key role in regulating cellular behavior by imperatively conditioning the processes of cell repair and regeneration [14].

Repair and regeneration of injured or diseased tissues are complex biological processes, during which many molecular, cellular, and tissue responses are involved [15], such as the proliferation and migration of epidermal cells towards the layer to be resealed.

The initial inflammatory response leads to the influx of macrophages and neutrophils, which release cytokines, growth factors, and nitric oxide, and induce nearby keratinocytes to migrate across the wounded epithelium [16].

While cytokines and growth factors can indeed stimulate keratinocyte migration, a much earlier stimulus of directed migration is triggered by wounding: the electrical field generated by the flow of current out of the wound [17].

Endogenous bioelectric fields (EBFs), detected in wounds and damaged tissues, can control cell differentiation, proliferation, and migration, enabling the robust restoration of the normal pattern after injury [14]. The recovering mechanism of the tissues is linked to the direct-current EBFs that are induced by the lesion. In fact, the EBFs are crucial in stimulating tissues regeneration [18]. When the production of EBFs in altered, the galvanotaxis processes are reduced and the recovering mechanism is no longer supported. This can be a conditioning and determining factor, among the various factors that can cause a delay in healing [19].

Different treatment strategies exist for the management of chronic wounds: some are invasive, such as wound debridement and skin substitute therapy; while others are noninvasive, such as compression bandaging, wound dressing, hyperbaric oxygen therapy, negative pressure therapy, ultrasound, and electrostimulation therapy (EST) [20]. The REAC TO-RPR treatment appears to be another option to promote fast and effective recovery of chronic wounds in a noninvasive way.

A clinical demonstration of this claim is represented by the cases described in this article.

Although the two lesions described have different origins, the manipulation of the endogenous bioelectric activity appeared to have led the two lesions to a common healing process that has proven to be rapid and refined compared to the classic expectations of healing with respect to these lesions.

## Conclusions

The REAC TO-RPR and regenerative tissue optimization (TO-RGN) treatments represent a powerful tool for neurobiological modulation, able to modulate, each for its own specific indications, the bioelectrical signals that control cell proliferation, migration, and differentiation. For this mechanism of action, they can affect the treatment of difficult wounds speeding up healing times, regardless of the cause that induced the injury. They favor a refined restoration of the damaged tissue, to allow the reappearance of the skin appendages also on the scarred area. Moreover, the speed and quality of the reparative process allow a rapid disappearance of pain in the BI case. Further studies can be useful to consolidate these results.
